# Proteomic analysis of B cells in peripheral lymphatic system reveals the dynamics during the systemic lupus erythematosus progression

**DOI:** 10.52601/bpr.2024.240045

**Published:** 2025-04-30

**Authors:** Liming Sun, Yuanyuan Yin, Yuqing Cao, Chunlei Chen, Yutong Guo, Zeming Cai, Jiarui Wu, Qingrun Li

**Affiliations:** 1 Key Laboratory of Systems Health Science of Zhejiang Province, School of Life Science, Hangzhou Institute for Advanced Study, University of Chinese Academy of Sciences, Hangzhou 310024, China; 2 School of Life Science and Technology, ShanghaiTech university, Shanghai 201210, China; 3 CAS Key Laboratory of Systems Biology, Shanghai Institute of Biochemistry and Cell Biology, Center for Excellence in Molecular Cell Science, Chinese Academy of Sciences, University of Chinese Academy of Sciences, Shanghai 200031, China

**Keywords:** Proteomics, B cells, Beripheral lymphatic system, SLE

## Abstract

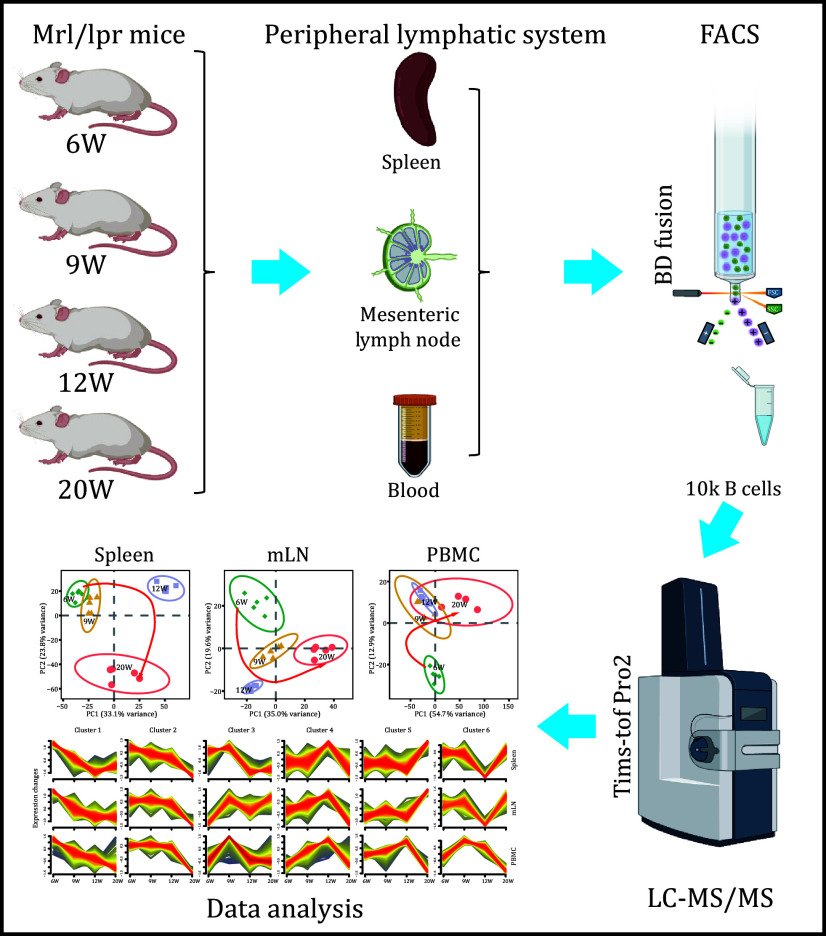

In this study, we conducted a comprehensive proteomic analysis of B cells from the spleen, mesenteric lymph nodes (mLN), and peripheral blood mononuclear cells (PBMC) in a time-course model of systemic lupus erythematosus (SLE) using female MRL/lpr mice. By combining fluorescence-activated cell sorting (FACS) and 4D-Data-Independent Acquisition (4D-DIA) mass spectrometry, we quantified nearly 8000 proteins, identifying significant temporal and tissue-specific proteomic changes during SLE progression. PBMC-derived B cells exhibited early proteomic alterations by Week 9, while spleen-derived B cells showed similar changes by Week 12. We identified key regulatory proteins, including BAFF, BAFFR, and NFKB2, involved in B cell survival and activation, as well as novel markers such as CD11c and CD117, which have previously been associated with other immune cells. The study highlights the dynamic reprogramming of B cell proteomes across different tissues, with distinct contributions to SLE pathogenesis, providing valuable insights into the molecular mechanisms underlying B cell dysregulation in lupus. These findings offer potential therapeutic targets and biomarkers for SLE.

## INTRODUCTION

Systemic lupus erythematosus (SLE) is an autoimmune disease characterized by its unclear pathogenesis and significant morbidity and mortality rates (Dorner *et al*. 2011). Although the etiology of SLE is multifaceted, the dysregulation of B cells is recognized as a pivotal factor in the pathogenesis of SLE (Merrill *et al*. 2010). Current therapeutic strategies for SLE frequently involve the depletion of B cells using specific antibodies (Kamburova *et al*. 2013) or chimeric antigen receptor (CAR)-modified T cells (Mougiakakos *et al*. 2021; Wilhelm *et al*. 2024). Various atypical B cell subtypes have been reported in individuals with SLE. CD27 was thought to be a classic marker of memory B cells; however, several studies identified that CD27 ^−^ memory B cells within the peripheral lymphatic system (PLS) of SLE patients (Ehrhardt *et al*. 2008; Jacobi *et* *al*. 2008; Wei *et al*. 2007). CD20 (Ms4a1), typically a marker of mature B cells, was absent in plasma B cells in SLE, which were a significant source of autoantibodies (Shan *et al*. 2017). In addition, CD11c, a marker of dendritic cells, was expressed in B cells from lupus patients, and this population of B cells was also expanded in common variable immunodeficiency disorder (CVID) (Isnardi *et al*. 2010; Saadoun *et al*. 2013). These findings underscored the profound dysregulation of B cells in SLE patients.

B cells were heterogeneous and dispersed across various tissues, making their study challenging. Current methodologies, including flow cytometry, cytometry by time of flight (CyTOF), and single-cell RNA sequencing (scRNA-seq), allowed for the analysis of B cells at the single-cell level (Gudjonsson *et al*. 2020; Wang *et al*. 2018; Wu *et al*. 2016). However, these techniques are limited to antibody-based targeting of specific proteins or low-depth, non-targeted transcriptomic analyses, which do not fully capture the comprehensive biological state of B cells. Moreover, next-generation sequencing-based researches could only elucidate changes at the RNA level (Gudjonsson *et al.* 2020; Wang *et al.* 2018), and the transcription levels did not always correlate accurately with protein expression levels (Yang *et al*. 2019). Consequently, investigating B cells from a proteomic perspective is crucial, yet such studies are currently lacking. Meanwhile, the temporal dynamics of B cell alterations during SLE pathogenesis remain poorly understood.

Here, we utilized MRL/lpr female mice as an animal model for SLE. B cells (CD45 ^+^CD19 ^+^) were sorted from the spleen, mesenteric lymph nodes (mLN), and peripheral blood mononuclear cells (PBMC) at various time points using flow cytometry. These cells were then subjected to a mass spectrometry-based 4D-Data-Independent Acquisition (4D-DIA) strategy to generate high-quality proteomic data. Our analysis revealed both similarities and differences in B cells from different organs, identifying PBMC-resident B cells as the first responders in SLE progression. Furthermore, our results demonstrated dynamic proteomic reprogramming in distinct B cell populations throughout the course of SLE pathogenesis.

## MATERIALS AND METHODS

### Animal model and experimental design

Female MRL/lpr mice at 6-week-old were purchased from Shanghai Lingchang Biotechnology Company. All mice were housed in SPF-level animal facilities under 22 °C and 12-hour light-dark cycles, provided with a standard mouse chow diet and water at the Animal Platform of the Center of Excellence in Molecular Cell Science. The mice were euthanized at Week 6, Week 9, Week 12 and Week 20, respectively. Each age group consisted of five mice. The animal care protocol was approved by the Institutional Animal Care and Use Committee (IACUC) of the Center of Excellence in Molecular Cell Science, Chinese Academy of Sciences.

### Tissue sampling

Mice were euthanized by intraperitoneal injection of sodium pentobarbital solution (60 mg/kg body weight). Blood was collected via abdominal aorta puncture. Mesenteric lymph nodes, spleen, and kidneys were excised for analysis. The cross-sectional area of lymph nodes and spleen was measured using Image J software (version Image J2, NIH Bethesda).

### Tissue and blood sample processing

The tissues were minced and homogenized in PBS, and centrifuged at 600 *g* for 5 min. The pellet was resuspended by RBC lysis buffer (BD pharmingen ^TM^), and filtered by a 100 μm nylon mesh filter. After centrifugation at 600 *g* for 5 min, the pellet was resuspended by FACS buffer (2 mmol/L EDTA, 0.5% BSA in PBS, pH 7.2). All these procedures were performed on ice or at 4 °C.

Fresh blood samples were collected in tubes containing anticoagulant, and obtained peripheral blood mononuclear cells (PBMC) using Ficoll density gradient centrifugation. Centrifugation was performed at 2000 r/min for 20 min at room temperature. The PBMC were resuspended by RBC lysis buffer, and filtered by a 100 μm nylon mesh filter. The subsequent process was similar to the tissue sample processing step.

### Fluorescence-activated cell sorting

Tissue cells and PBMC were resuspended in FACS buffer (2 mmol/L EDTA, 0.5% BSA in PBS, pH 7.2) at a volume of 1 mL per 10 ^7^ cells. Cells were incubated with fluorescently labeled antibodies CD45-APC (Thermo Fisher Scientific, 17-0451-82), CD45-APC-Cyanine7 (Thermo Fisher Scientific, A15395), CD19-FITC (Thermo Fisher Scientific, 45-0051-82), CD4-Pacific Blue (Thermo Fisher Scientific, MCD0428), and CD8a-PE (Thermo Fisher Scientific, 12-0081-82) on ice for 30 min. After incubation, cells were washed with cold 1× PBS to remove excess antibodies. Cells were resuspended in preparation for analysis and filtered through a 100 μm filter before running on the flow cytometer.

Flow cytometric analysis and sorting were performed using the BD FACSAria™ Fusion Cell Sorter (FACSAria Fusion, BD). CD45 ^+^CD4 ^+^ cells were identified as CD4 ^+^ T cells, CD45 ^+^CD8 ^+^ cells as CD8 ^+^ T cells, and CD45 ^+^CD4 ^−^CD8 ^−^CD19 ^+^ cells as B cells. B cells were sorted using purity mode and isolated into pre-prepared 0.6 mL centrifuge tubes containing 100 μL ACN. Each channel collected 2−5 × 10 ^4^ cells. Sorted B cells were stored at −80 °C. All flow cytometry data were generated and analyzed using FlowJo software (version 10.8.1, Leonard Herzenberg).

### Sample processing for proteomics

The sorted cell suspensions were shaken vigorously, and lyophilized in a vacuum freeze dryer. The dried samples were dissolved in 100 μL ATC buffer solution (100 mmol/L Ammonium Bicarbonate (NH _4_HCO _3_), 5 mmol/L Tris(2-carboxyethyl) phosphine hydrochloride (TCEP), 20 mmol/L 2-Chloroacetamide (CAA), pH 8), and mixed with 3 μL trypsin (Promega, VA9000). The samples were then incubated at 56 °C in a water bath for 1 h for digestion. Following digestion, peptides were desalted and purified using StageTips packed with three Empore™ SPE Disks C18 (3 m Purification). The peptides were eluted from the StageTips and subsequently lyophilized. The peptides were resuspended in 0.1% formic acid and subjected to mass spectrometry analysis.

### Liquid chromatography-tandem mass spectrometry analysis

All experiments were performed using a timsTOF Pro2 mass spectrometer (Bruker) coupled with a U3000 ultra-high performance liquid chromatography system (ThermoFisher). Samples were prepared using 0.1% formic acid ( *v*/ *v*) for effective sample loading. Tissue-derived peptides were separated using a 30-min gradient (0−24 min, 4%−23% B; 24−26 min, 23%−32% B; 26−28 min, 32%−95% B; 28−30 min, 95% B), both at a flow rate of 400 nL/min. Mobile phases A and B consisted of water with 0.1% formic acid ( *v*/ *v*) and acetonitrile with 0.1% formic acid ( *v*/ *v*), respectively. The mass spectrometer was coupled with U3000 with a capillary ion source (ion source voltage, 1.5 kV), and performed diaPASEF method to acquire spectra. The collision energy was linearly ramped from 59 eV at 1/K0 = 1.6 Vs/cm ^2^ to 20 eV at 1/K0 = 0.6 Vs/cm ^2^ based on ion mobility which was scanned from 0.59 to 1.6 Vs/cm ^2^ with a 100 ms ramp time.

### Database searching of MS data

DIA data was searched by Spectronaut™ (version 17, Biognosys Inc.) using directDIA+ with the library of Uniprot Mus musculus (10090) (version 2022_10, SwissProt,17201 sequences, reviewed). Default settings were used. In brief, the maximum allowed number of missed cleavage sites was two, PSM FDR, Peptide FDR and Protein Group FDR were set to 1%, and search peptide length was 7−52. Carbamidomethyl (C) was set as the fixed modification. Oxidation (M) and N-acetylation were set as the variable modifications. The tolerance of calibration search and main search were set to dynamic.

### Data normalization and missing value imputation for proteome data

After data acquisition, normalization was performed by median centering of quantitative information followed by log2 transformation. Missing values were imputed using a grouped approach based on tissue type and age. For data analysis, proteins identified in at least 50% of samples from any group (6 weeks, 9 weeks, 12 weeks, 20 weeks) are retained for each tissue type (spleen, mLN, PBMC). Missing values were imputed using half of the minimum value from the sample. Finally, the number of proteins retained for subsequent analysis in the spleen, mLN, and PBMC were 7398, 7316, and 7114, respectively.

### Differential protein and pathway enrichment analysis

ANOVA was used for differential expression analysis in the same tissues among different ages (6 weeks, 9 weeks, 12 weeks, and 20 weeks). The Student’s *t*-test was employed to analyze differential expression between specific age groups ( *e*. *g*., 6 weeks vs. 9 weeks, 6 weeks vs. 12 weeks, 6 weeks vs. 20 weeks, *etc*.). Additionally, gene ontology (GO) enrichment analysis of the significant proteins was performed using the DAVID bioinformatics database (https://david.ncifcrf.gov), with thresholds of FDR-adjusted *p*-value < 0.05 or other specified criteria to determine significantly regulated pathways.

### Statistical analysis

Statistical analyses and visualization were performed using R 4.1.2 and GraphPad Prism (version 9.5.0). Statistical significance testing, including Student's *t*-test, ANOVA, and Pearson correlation, was conducted using R. In GraphPad Prism, results are presented as mean ± SD. Group comparisons were assessed using one-sided *t*-test. The significance levels were denoted by asterisks: * *p* < 0.05, ** *p* < 0.01, *** *p* < 0.001, **** *p* < 0.0001.

## RESULTS

### Age-related changes in immune cell populations and morphological features in female MRL/lpr mice

**Figure 1:**
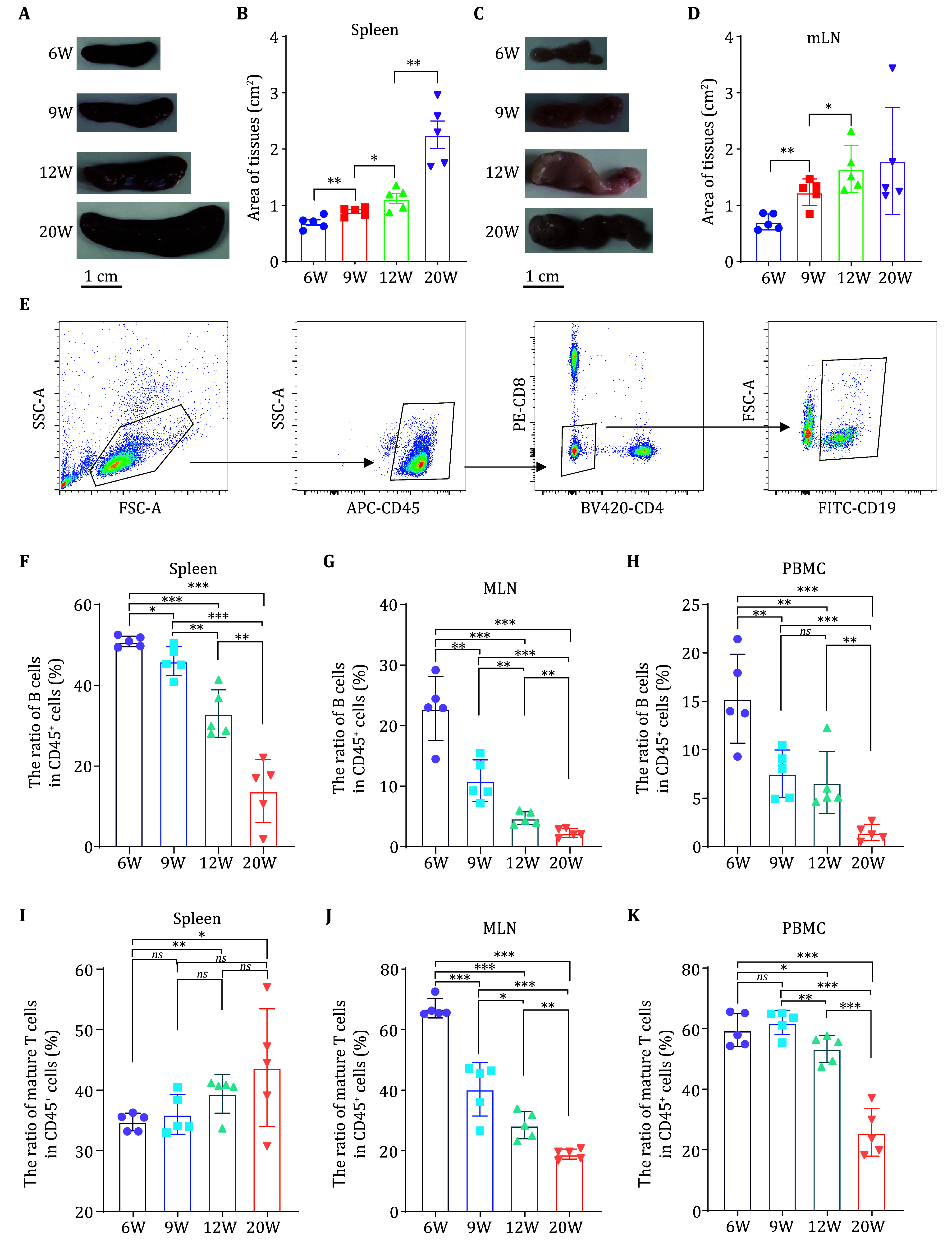
Age-related changes in immune cell populations and morphological features in female MRL/lpr mice. **A** Morphological changes in the spleen of female MRL/lpr mice at 6, 9, 12, and 20 weeks of age. **B** Changes in the spleen area of female MRL/lpr mice ( *n* = 5) at 6, 9, 12, and 20 weeks of age. **C** Morphological changes in the mesenteric lymph nodes of female MRL/lpr mice at 6, 9, 12, and 20 weeks of age. **D** Changes in the mesenteric lymph node area of female MRL/lpr mice ( *n* = 5) at 6, 9, 12, and 20 weeks of age. **E** Flow cytometry analysis method for B cells in the circulatory system and spleen of female MRL/lpr mice: First, in the FSC-A vs. SSC-A plot, forward scatter (FSC-A) and side scatter (SSC-A) are used to identify cell populations based on size and granularity. Then, in the APC-CD45 vs. SSC-A plot, gating on CD45 ^+^ cells is performed to identify leukocytes. Following this, the PE-CD8 vs. BV421-CD4 plot is used to identify CD4 ^+^ and CD8 ^+^ T cell populations with CD4 and CD8 markers. Finally, the FSC-A vs. FITC-CD19 plot is used to identify CD19 ^+^ B cells from the CD45 ^+^CD4-CD8- population. **F**− **H** Changes in the ratio of B cells (CD45 ^+^CD19 ^+^) among CD45 ^+^ T cells in the spleen ( **F**) mesenteric lymph nodes ( **G**) and peripheral blood mononuclear cells (PBMC) ( **H**) of female MRL/lpr mice ( *n* = 5) at 6, 9, 12, and 20 weeks of age. **I**− **K** Changes in the ratio of mature T cells (CD45 ^+^CD4 ^+^ or CD45 ^+^CD8 ^+^ cells) among CD45 ^+^ T cells in the spleen ( **I**) mesenteric lymph nodes ( **J**) and peripheral blood mononuclear cells (PBMC) ( **K**) of female MRL/lpr mice ( *n* = 5) at 6, 9, 12, and 20 weeks of age

To study the proteomic changes in B cells during the pathogenesis of lupus erythematosus, we employed female MRL/lpr mice at different ages (6 weeks, 9 weeks, 12 weeks, and 20 weeks). Spleen, mesenteric lymph nodes (mLN), and peripheral blood mononuclear cells (PBMC) were collected for analysis. Consistent with previous reports, significant enlargement of the spleen and lymph node was observed in the MRL/lpr mice ( Figs. 1A and 1C). The spleen and mLN continued to enlarge as the disease progressed, however, after 12 weeks, the size of these mLN did not show significant changes ( Figs. 1B and 1D).

To obtain pure B cell populations and exclude contamination by other cells, we used flow cytometry with a gating strategy targeting CD45 ^+^CD4 ^−^CD8 ^−^CD19 ^+^ cells ( Fig. 1E and supplementary Figs. S1−S3). Analysis revealed that B cells in the spleen, mLN, and PBMC exhibited a progressive decline in the population of CD45 ^+^ cells over time. Specifically, the ratio of B cells to CD45 ^+^ cells in the spleen, mLN, and PBMC decreased as the disease advanced. Notably, while the splenic B cell ratio to CD45 ^+^ cells was reduced to half of its 6-week value by Week 20, the B cell ratios in the mLN, and PBMC declined to below 50% by Week 9 ( Figs. 1F−1H). This suggests that the B cells in the mLN and PBMC undergo more pronounced abnormalities earlier in the disease course, which is consistent with the observed morphological changes.

As SLE progressed in MRL/lpr mice, the generation of “double-negative T cells” within the peripheral immune system influenced the proportion of mature T cells (CD45 ^+^CD4 ^+^ and CD45 ^+^ CD8 ^+^) (Li *et al*. 2020). We found that the ratio of mature T cells to CD45 ^+^ cells varied with disease progression across different organs. In the spleen, this ratio showed a slight increasing trend with age. In contrast, the PBMC exhibited a decrease in mature T cell rations starting at Week 12, and the mLN demonstrated a significant reduction as early as Week 9 ( Figs. 1I−1K). These findings highlight that immune organs respond to SLE progression at different time points, with the mesenteric lymph nodes being the most sensitive to disease onset. In this part, we summarized the differences in histomorphology and proportions of acquired immune cells in immune organs of MRL/lpr mice at different disease stages, demonstrating that mesenteric lymph nodes are particularly sensitive to SLE progression.

### Comparative proteomic analysis of B cells across spleen, mesenteric lymph nodes, and PBMC in MRL/lpr female mice

**Figure 2:**
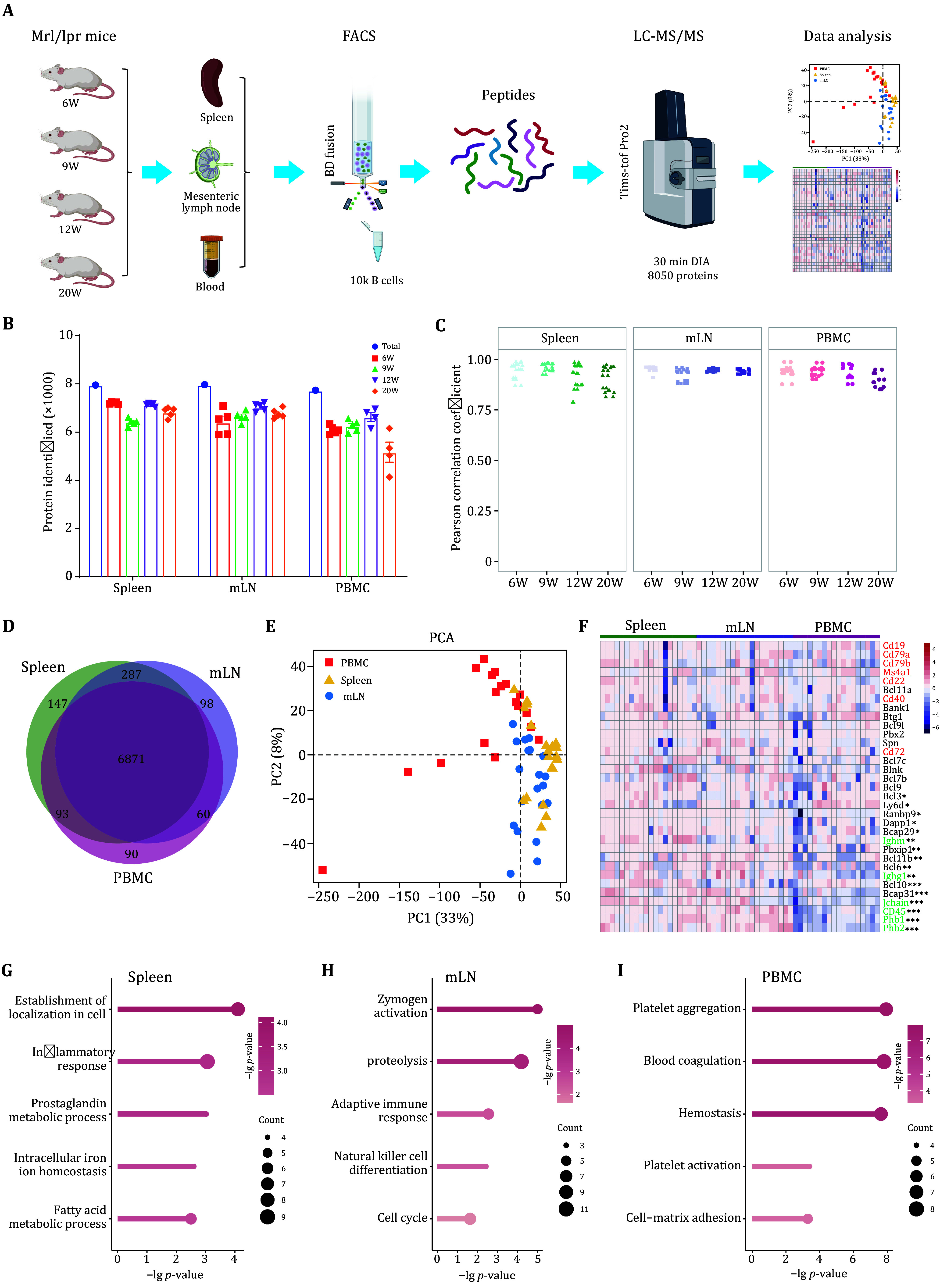
Comparative proteomic analysis of B cells across spleen, mesenteric lymph nodes, and PBMC in MRL/lpr female mice. **A** Workflow for isolating B cells and performing proteomic analysis from the spleen, mesenteric lymph nodes, and peripheral blood of MRL/lpr female mice. **B** Quantification of the number of proteins detected in B cells from the spleen, mesenteric lymph nodes, and PBMC of mice at different ages. **C** Pearson correlation scatter plots of B cell samples from the same age mice in three different tissues (spleen, mesenteric lymph nodes, PBMC). **D** Venn diagram showing overlapping and tissue-specific proteins in the spleen, mesenteric lymph nodes, and PBMC. **E** PCA plot of proteomic profiling with total proteins in spleen, mesenteric lymph nodes, and PBMC. **F** Heatmap showing expression of B cell markers or specific proteins in spleen, mesenteric lymph nodes, and PBMC. The red font represents classic B cell markers. Proteins marked with an asterisk (*) in the upper right corner show significant differences between the three groups: * *p* < 0.05, ** *p* < 0.01, *** *p* < 0.0001. **G**− **I** GO-BP enrichment analysis of tissue-specific or highly expressed proteins in spleen ( **G**) mesenteric lymph nodes ( **H**) and peripheral blood mononuclear cells (PBMC) ( **I**)

To further investigate the protein expression signatures of B cells within the immune system during SLE disease progression, we sorted 10,000 CD45 ^+^CD19 ^+^ B cells from the spleen, mLN, and PBMC separately using flow cytometry. The sorted cells were then lysed, digested, and desalted for mass spectrometry-based 4D-DIA ( Fig. 2A) analysis.

In total, we quantified 8050 proteins, with more than 6000 proteins consistently quantified in each replicate, except for the 20-week PBMC samples. Nearly 8000 proteins were identified in each organ ( Fig. 2B). Pairwise correlation analysis of biological replicates from mice of the same age across spleen, mLN, and PBMC Tissues yielded Pearson correlation coefficients ranging from 0.79 to 0.99, with a median of 0.95 ( Fig. 2C), indicating a strong positive correlation between biological replicates (6 weeks, 9 weeks, 12 weeks, and 20 weeks). After median normalization and imputation of missing values, 7646 proteins were retained for subsequent data analysis. Specifically, 7398, 7316, and 7114 proteins were included in the B-cell proteomics analysis (supplementary Table S1). Among these, 6871 proteins were consistently quantified across all three B cell populations ( Fig. 2D).

Principal component analysis (PCA) of these co-identified proteins revealed that B cells from various organs exhibited distinct tissue-specific characteristics. The first principal component primarily separated PBMC-derived B cells from those derived from the spleen and mLN ( Fig. 2E). To validate the reliability and accuracy of our proteomic data, we screened the data for B-cell-specific expressed markers (supplementary Table S2). Common biomarkers widely used in flow cytometry and cell annotation in single-cell RNA-sequencing were consistently identified across all B cell populations with no significant differential expression, including Cd19, Ms4a1 (Cd20), Cd22, Cd79a, Cd79b, *etc*. However, Ighm and Jchain showed differential expression (ANOVA FDR < 0.05). Additionally, Phb1 and Phb2 were reported to regulate the expression of Ighg1 in B cells, and showed the lowest expression levels in PBMC-derived B cells ( Fig. 2F).

We defined tissue-specific resident B cell signature proteins as those exclusively identified in one B cell population or significantly up-regulated by more than 3-fold in one specific tissue ( Fig. 2D, supplementary Figs. S4A−S4C and Table S3). GO analysis revealed that these signature proteins were enriched in distinct biological processes corresponding to each tissue’s function. Spleen-derived B cell signatures were involved in the establishment of localization in cells, inflammatory response, and fatty acid metabolic process. In contrast, mLN-derived B cells were enriched in zymogen activation, adaptive immune response and cycle, while proteins in PBMC-derived B cells were enriched in platelet aggregation, blood coagulation, and platelet activation ( Figs. 2G−2I). These enriched processes reflect the specific biological functions of the organ. For instance, the platelet-related functions were integral to blood, and proteins involved in this process, including Tubb1, Itgb3, Myl9, Fga, Fgb, and Fgg, were significantly overexpressed in PBMC-derived B cells (supplementary Fig. S4D).

In summary, we established a high-quality B cell proteomic database by combining flow cytometry with mass spectrometry. This enabled the annotation of tissue-specific proteins in B cells, providing valuable insights into the distinct proteomic landscapes of B cells from the spleen, mLN, and PBMC during SLE progression in MRL/lpr female mice.

### Temporal dynamics of the B cell proteomes from the spleen, mesenteric lymph nodes, and PBMC during the onset and progression of SLE

**Figure 3:**
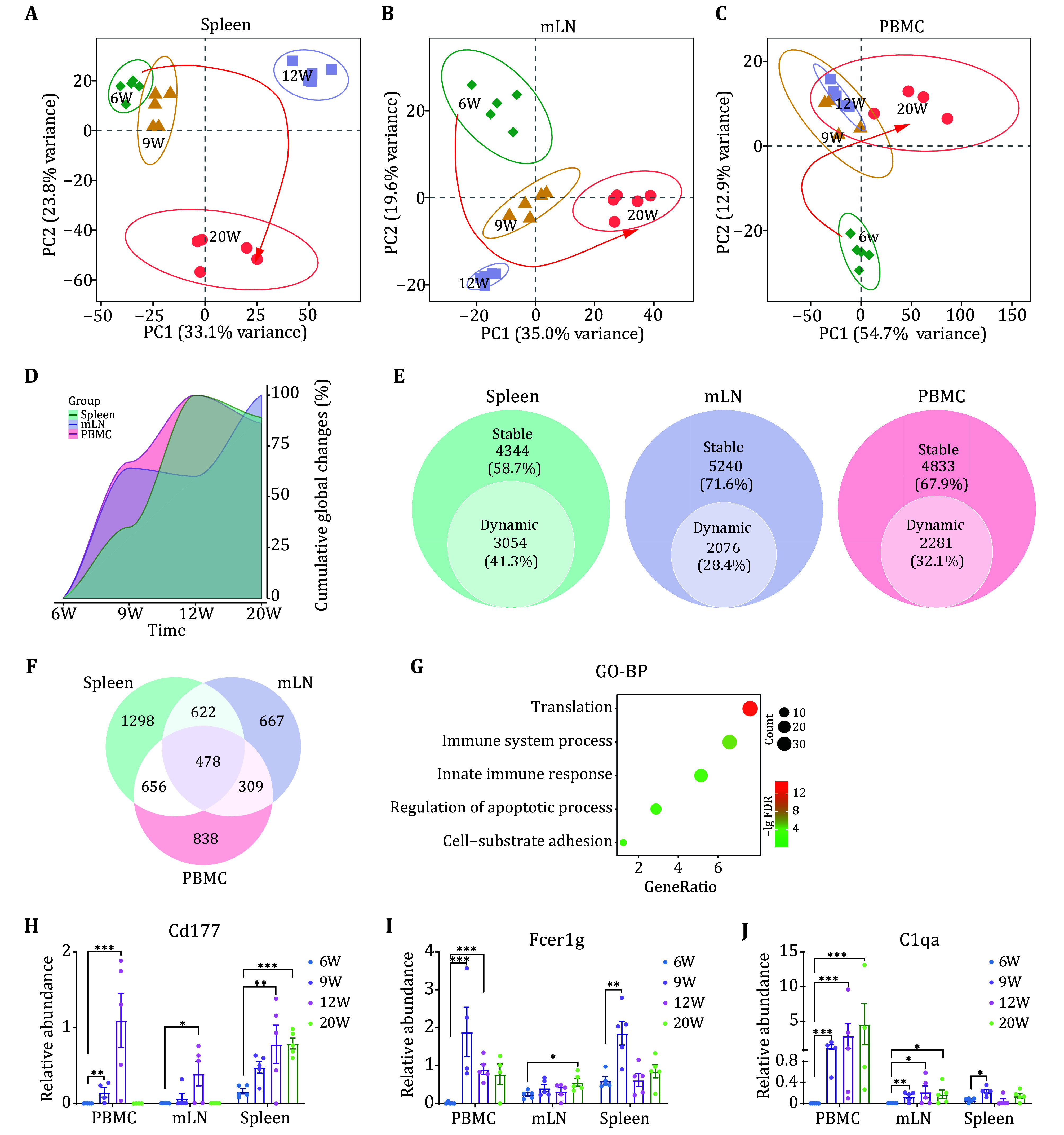
Temporal dynamics of the B cell proteomes from the spleen, mesenteric lymph nodes, and PBMC during the onset and progression of SLE. **A**− **C** Principal component analysis of B cell proteomes from the spleen ( **A**), mesenteric lymph nodes ( **B**), and PBMC ( **C**) during the onset and progression of SLE. Green diamonds, yellow triangles, purple squares, and red circles represent mice at 6, 9, 12, and 20 weeks, respectively. **D** Temporal dynamics of global changes in B cell proteomes from the spleen, mesenteric lymph nodes, and PBMC during the onset and progression of SLE. The percentage of dynamically regulated proteins was calculated at each time point and scaled to the maximum percentage in the spleen, mesenteric lymph nodes, and PBMC. Locally weighted scatterplot smoothing (LOESS) was used to fit the percentage data at each time point. **E** Fraction of proteins with significant changes at any time point during the onset and progression of SLE in the spleen, mesenteric lymph nodes, and PBMC. **F** Venn diagram showing the overlap of dynamic proteins from Panel E. **G** GO-BP enrichment analysis of the 478 overlapping proteins identified in the spleen, mesenteric lymph nodes, and PBMC from Panel F. **H**− **J** Expression changes of key proteins Cd177 ( **H**), Fcer1g ( **I**), and C1qa ( **J**) in B cells from the three sources at 6, 9, 12, and 20 weeks

PCA analysis of the three organ-derived B cells revealed clear time-dependent separation of the proteomic profiles, suggesting significant differences in the behavior of B cells across organs during SLE disease progression. Specifically, spleen-derived B cells showed a minimal difference between 6 weeks and 9 weeks, but exhibited drastic changes starting at 12 weeks, which were further pronounced at 20 weeks ( Fig. 3A). In contrast, mLN-derived B cells maintained a uniform progression trend without drastic changes at any specific time point ( Fig. 3B). However, PBMC-derived B cells underwent drastic alterations as early as 9 weeks, followed by slower progression thereafter ( Fig. 3C).

To understand the molecular events sequence and temporal dynamics of B cells in SLE disease progression within the peripheral lymphatic system, we carefully examined the timing and magnitude of proteomic changes in B cells. Consistent with the PCA results, spleen-derived B cells exhibited delayed proteomic alternations compared to other B cell populations. The expression trends of B cells from the three organs formed distinct time series, reaffirming the differential impact of SLE on B cells in each organ over time ( Fig. 3D, supplementary Figs. S5A−S5C).

Next, we assessed the magnitude of protein expression changes in B cells from three different sources during SLE progression. We found that spleen-derived B cells experienced the highest degree of proteomic regulation, followed by PBMC-derived B cells, and mLN-derived B cells showed the least regulation ( Fig. 3E). In detail, a systematic comparison of differentially regulated proteins showed that 41.3% of the spleen-derived B cells were dynamically regulated over time, followed by 32.1% of PBMC-derived B cells, while only 28.4% of mLN-derived B cells were regulated ( Fig. 3E). To find similarities among B cell proteins from three different sources that were regulated during disease progression, we considered proteins that were differentially regulated in all types of B cells. Our results showed that only 478 proteins were screened ( Fig. 3F, supplementary Table S4), and these proteins were primarily involved in biological processes such as translation, immune system process, apoptotic process, *etc*. ( Fig. 3G). These findings suggest enhanced protein synthesis and activation of immune functions in B cells, ultimately leading to apoptosis during SLE progression.

Focusing on proteins involved in immune system processes, we identified several key regulators ( Figs. 3H−3J). Cd177, a specific marker of neutrophil activation (Kim *et al*. 2021), was also detected in regulatory T cells, and blocking the expression of Cd177 could reduce the inhibitory immune activity of Treg and lead to decreased tumor growth in mice (Kim *et al.* 2021), suggesting the function of promoting inflammatory. In our data, Cd177 was detected to be expressed in B cells and significantly overexpressed by 12 weeks, indicating B cell activation ( Fig. 3H). Fcer1g was a shared subunit of multiple Fc receptors that activated the SYK-RAS-MAPK pathway and the NF-kappaB pathway through its intracellular immunoreceptor tyrosine-based activation motif (ITAM) (Bruhns and Jonsson 2015), which were critical signaling pathways in B cell activation (Burger and Wiestner 2018). This protein was significantly upregulated in B cells in both PBMC- and spleen-derived B cells at Week 9 compared to Week 6 ( Fig. 3I), suggesting early activation of B cells in these organs. In addition, the activation of the complement system in LN was associated with the onset of SLE (Walport 2001). We observed the induction of C1qa in all types of B cells at Week 9, further suggesting activation of the complement system in B cells of the peripheral blood and lymphatic system ( Fig. 3J).

Taken together, our findings indicated that PBMC-derived B cells were the earliest responders to SLE disease signals, followed by spleen-derived B cells. Throughout the SLE disease progression, B cells in PBMC and spleen exhibited substantial proteomic regulation, highlighting their critical roles in SLE pathogenesis.

### Temporal dynamics of B cell proteome changes and key regulatory factors in SLE progression

To model the dynamics of B cell proteome changes upon the SLE progression, we identified the differential expression proteins in B cells from each source over time using ANOVA (FDR < 0.05). Subsequently, we performed the fuzzy c-means algorithm to partition all differential expression proteins into six optimal clusters based on their temporal expression profiles. A total of 3711, 1093, and 2069 differentially expressed proteins were identified in B cells of the spleen, mLN, and PBMC, respectively (supplementary Table S5).

**Figure 4:**
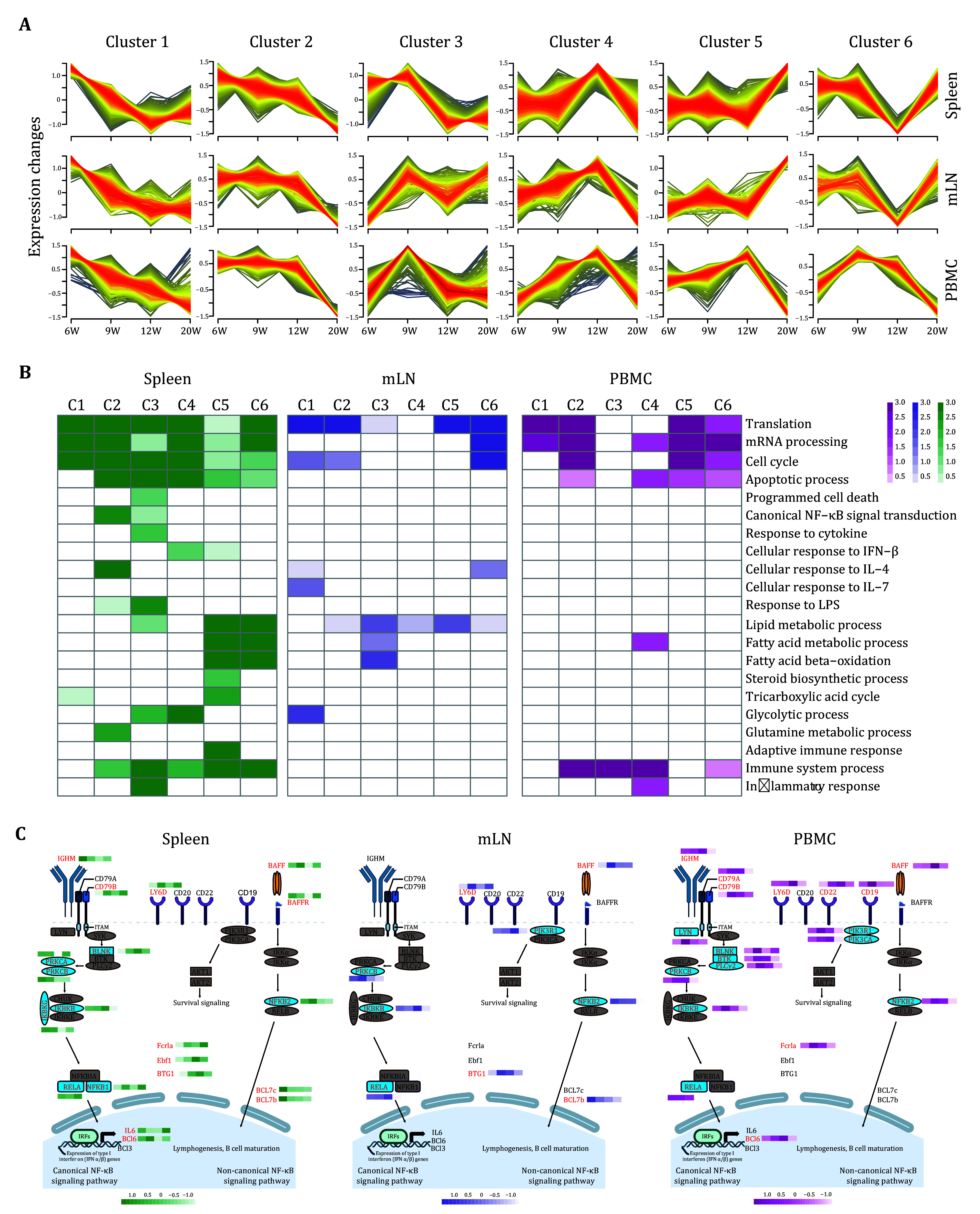
Temporal dynamics of B cell proteome changes and key regulatory factors in SLE progression. **A** Fuzzy c-means clustering of differential B cell proteins in the spleen, mesenteric lymph nodes and PBMC. The number of differential proteins identified was 3711 in the spleen, 1093 in mesenteric lymph nodes, and 2069 in PBMC. **B** GO-BP enrichment analysis of each cluster from Panel A for spleen, mesenteric lymph nodes, and PBMC. **C** Diagram of B cell survival, proliferation, maturation, and B cell receptor signaling pathways, with expression changes of relevant proteins during SLE pathogenesis. Red text indicates the results of ANOVA over time, with P-FDR < 0.05, Gray text indicates a lack of significance

The clustering results demonstrated that the six expression trends in spleen and mLN were similar; however, the biological processes enriched in each cluster presented some differences ( Figs. 4A and 4B). In mLN-derived B cells, biological processes related to lipid metabolism, including lipid metabolic process, fatty acid metabolic process, fatty acid β-oxidation, and steroid biosynthetic process were primarily associated with Cluster 3. In contrast, in spleen-derived B cells, these processes were enriched in Clusters 5 and 6 ( Fig. 4B). In addition, clusters in the spleen were enriched with biological processes uniquely identified within the spleen, such as cellular response to IL4, IL7, and IFN-β, immune system process, and inflammatory response ( Fig. 4B).

The trends of Clusters 1−4 in PBMC were similar to those observed in the spleen and mLN, while Cluster 5 and Cluster 6 mirrored the trends of Cluster 2 in PBMC ( Fig. 4A). The processes enriched in Cluster 5 and Cluster 6 were identical to those in Cluster 2, indicating a significant decrease in immune system process of B cells at Week 20. This decrease may be related to the reduction of B cells in PBMC ( Fig. 4B).

Finally, we identified key proteins involved in B cell survival, proliferation, maturation, and B cell receptor (BCR) signaling pathway during the SLE pathogenesis. The B cell activation factor (BAFF), produced by hematopoietic cells, plays an essential role in B cell activation and survival (Moore *et al*. 1999). BAFF has been reported to be expressed at the mRNA level in B cells of the spleen and bone marrow (Stadanlick *et al*. 2008). In the current study, we confirmed that B cells in the spleen, mLN and PBMC were able to express BAFF at the protein level, and B cells in the spleen and mLN showed the highest expression of BAFF at 9 weeks during the progression of SLE, while at 12 weeks in PBMC ( Fig. 4C).

BAFFR, the receptor of BAFF, mediated B cell survival and maturation signals through the non-canonical NF-κB signaling pathway (Mockel *et al*. 2021), was significantly regulated only in spleen-derived B cells during SLE progression, with an expression trend similar to BAFF. NFKB2, the key protein in the non-canonical NF-κB signaling pathway, was regulated in B cells from all three sources, and exhibited the lowest expression at 20 Weeks ( Fig. 4C), which might lead to the loss of B cell survival signals and subsequent apoptosis.

We also examined the BCR complex and other classic B cell markers. Only Ly6d was significantly regulated in B cells in mLN; IGHM, Cd79b, and Ly6d were significantly regulated in spleen-derived B cells; and B cells in PBMC had the most regulated markers, including IGHM, Cd79a/b, Ly6d, Cd19, and Cd22 ( Fig. 4C). The downstream of activated BCR signaling involved the SYK-BTK-PKC signaling pathway followed by the NF-κB pathway. Our data identified all key proteins within these signaling pathways, with most being significantly regulated during SLE progression in spleen and PBMC-derived B cells, although without a clear rhythm ( Fig. 4C). The expression levels of all differentially expressed proteins in PBMC-derived B cells were the lowest at week 20 ( Fig. 4C). In contrast, spleen-derived B cells exhibited a more disordered pattern of protein expression ( Fig. 4C).

## DISCUSSION

In this study, we present a comprehensive global proteomic analysis of the B cells within the PLS during the progression of SLE pathogenesis by a combination of the FACS and 4D-DIA mass spectrometry. We successfully constructed a high-quality B cell proteome database and conducted an in-depth comparison of B cells derived from the spleen, mLN, and PBMC. Our results demonstrated that the proteome of PBMC-derived B cells underwent significant changes as early as Week 9 of SLE progression, whereas spleen-derived B cells showed substantial proteomic alternations by Week 12 compared to their healthy state at 6 weeks (Reilly and Gilkeson 2002). Proteins involved in immune system processes were significantly regulated across all three B cell populations, revealing the pivotal role of B cells in the progression of female MRL/lpr mice. Finally, the dynamic of BCR signaling and its downstream NF-κB pathway varied among B cells from different organs over time, suggesting distinct contributions of B cells from each organ to SLE progression.

Our proteomic data not only identified most of the well-documented B cell markers, such as CD19, CD20, CD79a, CD79b, and IGHM, but also revealed the expression of proteins traditionally associated with other immune cell types, including CD11c (Itgax) and CD117. CD11c was typically recognized as a dendritic cell marker; however, it had been reported to be expressed in B cells of individuals with common variable immunodeficiency disorders (Isnardi *et al.* 2010; Saadoun *et al.* 2013). Similarly, CD117 was commonly expressed heterogeneously by neutrophils (Kissel *et al*. 2001; Stroncek 2007), and had also been confirmed on the surface of Treg cells (Kim *et al.* 2021). Our findings confirmed the expression of CD117 in B cells within the context of SLE, expanding the known marker repertoire for B cells. Beyond CD family molecules, our data uncovered numerous other membrane proteins and transcription factors not previously reported in B cells, thereby providing a robust database for a more comprehensive understanding of B cell biology.

Flow cytometry analysis showed a continuous decline in the proportion of B cells across all three organs as SLE progressed. Intriguingly, the apoptosis-related proteins did not trace significant activation over time. This observation may be attributed to the genetic background of the MRL/lpr mouse model, which harbored a monogenic mutation in the Fas receptor gene (Richard and Gilkeson 2018; Wu *et al*. 1993). This mutation impairs apoptosis in B cells (Richard and Gilkeson 2018), suggesting that the observed decrease in B cell proportions is likely due to the proliferation of other cell types, such as CD3 ^+^CD4 ^−^CD8 ^−^ cells (Mountz *et al*. 1994; Zhou *et al*. 1993), rather than apoptosis.

While female MRL/lpr mice are a widely used model for studying SLE, it is important to acknowledge certain limitations. Disease progression can vary among individual mice at the same age (Reilly and Gilkeson 2002), leading to heterogeneity in protein expression trends, particularly within the BCR signaling pathway. In addition, although MRL/lpr mice develop a large number of lupus-associated autoantibodies ( *e*. *g*., ANA, Anti-dsDNA) and complications such as arthritis and skin rash (Alexander *et al*. 1983; Eisenberg *et al*. 1978), the Fas mutation primarily cause lymphadenopathy in humans without inducing severe SLE symptoms (Rensing-Ehl *et al*. 2014). Moreover, the pathogenesis of SLE is highly complex, and female MRL/lpr mice do not fully recapitulate human lupus. Consequently, our findings may be species-specific and should be interpreted with caution when extrapolating to human SLE.

## CONCLUSION

In conclusion, to investigate the temporal dynamic changes of B cells at the proteomic level during the pathogenesis of SLE, we selected female MRL/lpr mice as the animal model. We established four-time points: 6 weeks, 9 weeks, 12 weeks, and 20 weeks, and B cells were isolated from the spleens, mesenteric lymph nodes, and PBMC of mice at each time point. Subsequently, the peptide samples prepared from these B cells were analyzed using a mass spectrometry-based 4D Data-Independent Acquisition (4D-DIA) strategy, which generated high-quality proteomic data. We observed that B cells from different organs exhibited both similarities and tissue specificity. Notably, B cells from PBMC responded first during the progression of SLE, followed by those from the spleen. Furthermore, our data revealed dynamic changes in key proteins related to B cell survival, proliferation, maturation, B cell receptor (BCR) complex, and BCR signaling pathways. Our results indicate that there is temporal dynamic proteomic reprogramming among different B cell populations during the onset and progression of SLE.

## Conflict of interest

Liming Sun, Yuanyuan Yin, Yuqing Cao, Chunlei Chen, Yutong Guo, Zeming Cai, Jiarui Wu and Qingrun Li declare that they have no conflict of interest.
